# Impact of treatment delays on future survival outcomes in non-surgical patients with esophageal cancer in Shandong Province, China

**DOI:** 10.3389/fonc.2024.1445267

**Published:** 2024-07-19

**Authors:** Yindi Sun, Pei Zhang, Dongqian Zhang

**Affiliations:** ^1^ Oncology Department, Zibo Central Hospital, Zibo, China; ^2^ The Sixth Department of Oncology, The Fourth People’s Hospital of Zibo, Zibo, China

**Keywords:** esophageal cancer, delay, overall survival, cancer-specific survival, China

## Abstract

The treatment process of tumors in surgical patients is typically prompt and efficient, whereas non-surgical patients are more prone to treatment delay due to various factors. However, the relationship between treatment delay and survival outcomes in non-surgical Esophageal cancer (EC) patients has received limited study. This study aims to evaluate the impact of waiting time from diagnose to treatment on survival outcomes among non-surgical EC patients in Shandong Province, China. Over a 20-year follow-up period, a total of 12,911 patients diagnosed with EC and not receiving surgical intervention were identified from 2000 to 2020. The Kaplan-Meier methodology was employed to determine overall survival (OS) and cancer-specific survival (CSS). Univariate and multivariate Cox regression analyses were performed to evaluate the impact of treatment delays on future outcomes. The nonlinear association between waiting time and survival outcomes was investigated using restricted cubic spline (RCS) functions. The average delay in initiating EC treatment from the initial medical consultation for symptoms of EC was 1.18 months (95%CI=1.16-1.20). Patients with a long delay (≥3 months) in treatment demonstrated significantly lower rates of 1-, 3-, and 5-year OS and CSS compared to those with a brief delay in treatment initiation. A long delay in EC treatment independently associated with an increased risk of mortality from all causes and cancer. The association between waiting time and both all-cause and cause-specific mortality illustrated a pronounced J-shaped pattern. The prolong delay in treatment initiation significantly impacts the OS and CSS outcomes for non-surgical EC patients. Timely administration of treatment has the potential to enhance survival outcomes in patients with EC who are ineligible for surgery, including those in advanced stages without surgical options available.

## Introduction

The global burden of cancer presents a significant public health challenge and holds a crucial position among worldwide diseases (1–3). EC ranks eighth in terms of incidence globally, while it stands at the sixth spot for cancer-related mortality on a global scale (4).

Delaying cancer treatment can have a detrimental impact on patient outcomes. Previous meta-analyses have consistently provided evidence supporting a significant correlation between delay and increased mortality (5–8). To effectively design cancer care systems, pathways, and models of care that yield equitable and cost-effective outcomes, it is necessary to comprehend the implications of delays on mortality rates (9). The importance of understanding the implications of treatment delays on outcomes has become increasingly prominent in light of the coronavirus 2019 (COVID-19) pandemic. Many countries have experienced disruptions in elective cancer surgeries and radiotherapy, as well as reductions in the utilization of systemic treatments due to healthcare resource reallocation for pandemic preparedness (10–14). The diagnosis of cancer patients without surgical options, on the other hand, carries a significant risk of treatment delays, which can have an adverse impact on their prognosis. This is primarily due to the fact that surgery is often considered the primary treatment modality for many types of cancer, particularly those that are localized and have not yet metastasized (15). Furthermore, the financial implications of cancer treatment can also contribute to delays in treatment for those without surgical options. Surgical treatment is often covered by insurance, while other treatment modalities, such as chemotherapy or radiation therapy, may not be fully covered or may require higher out-of-pocket expenses in developing countries (16). Consequently, this can result in delays in the initiation of treatment.

The objectives of this study are to investigate the impact of time from diagnosis to treatment on OS and CSS in non-surgical patients with EC. Additionally, we aim to evaluate the association between the duration from diagnosis to treatment and the risk of all-cause mortality as well as cancer-specific mortality.

## Methods

### Data source

**Figure 1:**
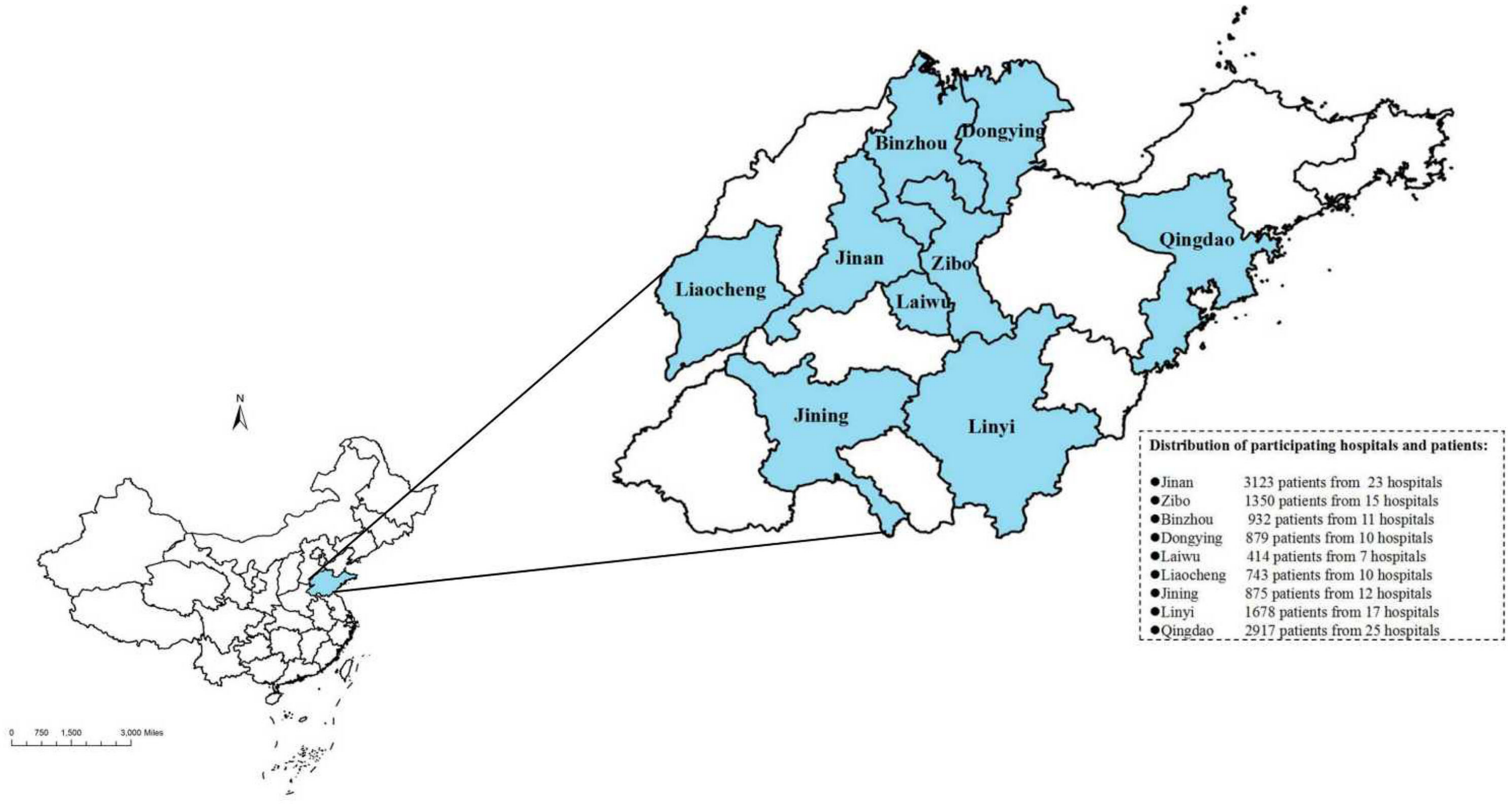
The distribution of participanting hospitals and patiens in Shandong, China. (ArcGIS 10.2 for Desktop software, Environmental Systems Research Institute Inc., USA, https://www.esri.com).

Data on patients were collected from 130 hospitals across 9 cities in Shandong Province, China. The distribution of participating hospitals is shown in **Figure 1**. The data collection period spanned from 2000 to 2020. The present study adhered to the checklist provided by the Strengthening the Reporting of Observational Studies in Epidemiology (STROBE) statement (17).

### Patient selection

The study recruited patients who met the ICD-O-3/WHO 2008 criteria for a diagnosis of “Esophagus” and exhibited a “Malignant” behavior code for the primary neoplasm. Cases without surgical intervention within the period from 2000 to 2020 were selected. In our study, we meticulously screened and excluded cases that lacked essential details, including the waiting time for treatment, other clinical information, and follow-up data. Due to the limited sample size and concerns regarding validity, patients who experienced treatment delays exceeding 6 months were excluded from the analysis (18).

## Variables of interest

### Demographic information

The information encompasses demographic characteristics, including sex, age at diagnosis, marital status at diagnosis, household income, living areas, and survival months. Marital status was divided into couple and single categories, which included separated, divorced, widowed, and never married individuals. Median household income per year was categorized into three groups, low level (≤50,000 Yuan), middle level (50,000-70,000 Yuan) and high level (≥70,000 Yuan). The living areas were classified into metropolitan counties and nonmetropolitan counties.

### Tumor-related information

The information encompasses tumor-related data, including primary site, differentiation, grade classification, histological characteristics, as well as radiotherapy and chemotherapy treatment history. The primary site of EC was categorized as the upper third, middle third, lower third, and other sites. The differentiation grade: Grade I represents well-differentiated, Grade II indicates moderately differentiated, Grade III signifies poorly differentiated and Grade IV denotes undifferentiated. The treatment history of radiotherapy and chemotherapy was dichotomized as either present or absent. The histology of EC was classified into squamous cell carcinoma, adenocarcinoma, and other group carcinoma.

### Definitions of delays to treatment

The lack of guidelines or consensus panels to establish thresholds for delayed treatment following a diagnosis has led to the adoption of specific time intervals. As such, we have defined treatment delays as the number of days between the initial medical consultation for symptoms of EC and the initiation of treatment. A delay of ≥1 month from diagnosis to the initiation of initial treatment is considered a treatment delay, while a delay of ≥3 months is classified as a severe treatment delay, in accordance with previous studies (18–20). According to the monthly records, patients were categorized into three groups based on the duration between diagnosis and treatment: brief treatment (within 1 month after diagnosis), moderate delay (1-2 months delay), and long delay (≥3 months) group.

### Definition of outcomes

In terms of the clinical outcome, OS and CSS were defined as the primary and secondary outcome, respectively. OS is a widely accepted measure in oncology trials that provides a comprehensive overview of patient survival throughout the study, quantifying the duration from treatment initiation to death from any cause. Conversely, CSS focuses specifically on evaluating treatment efficacy by considering only the survival of patients with the specific type of cancer under investigation. This outcome holds particular significance when assessing potential impacts on overall patient survival.

### Ethics statement

The study strictly followed the ethical principles delineated in the 1964 Helsinki Declaration along with its subsequent amendments or comparable ethical guidelines. Ethical approval in this study was obtained from the Institutional Review Board (IRB) of Zibo Central Hospital (IRB2022-00001032).

### Statistical analysis

The chi-square test was utilized to compare the variations among different levels of each factor. The Kaplan-Meier method was employed to analyze the 1-, 3- and 5-year OS and CSS rates, as well as the median survival time. To assess differences between the survival curves, the log-rank test was conducted. Univariate and multivariable survival analyses were performed using Cox proportional hazard regression models to calculate hazard ratios (HRs) and their corresponding 95% confidential intervals (CIs). A multivariable Cox proportional hazard regression model was constructed by incorporating risk factors that showed a significance level below 0.10 in univariate analysis. The sensitivity and specificity were evaluated by employing the receiver operating characteristic (ROC) curve, specifically quantifying the area under the curve (AUC). AUC values greater than 0.7 are considered high level fits. The RCS functions were employed to examine the non-linear association between treatment delay and both all-cause mortality and cancer-specific mortality (21, 22). The final cox models and confounders were employed in this analysis to explore the nonlinear association. In accordance with previous research recommendations, we chose the median time of treatment delay as the reference value for all analyses concerning nonlinear association. The incorporation of 3 knots into the models optimized the fit of nonlinear curves, thereby preventing accuracy reduction caused by over-fitting (23).

The analyses were performed using R software version 4.3.1. All statistical tests followed a two-tailed approach, and a significance level of P less than 0.05 was considered statistically significant.

## Results

### Characteristics of patients

During the study period, a total of 47,714 non-surgical patients diagnosed with EC were included in the analysis. Of these patients, data on waiting time from diagnosis to treatment was missing for 13,909 individuals and other important information was absent for 20,894 individuals. Ultimately, the study cohort consisted of 12,911 patients with an average age of 66.5 (range: 66.3-66.7) between the years of 2000 and 2020. Up to 2020, a total of 12,334 patients had died; among them were 10,907 deaths attributed to EC. The characteristics of EC patients, stratified by the duration between diagnosis and treatment, are presented in **Table 1**. Among all the patients, 22.1% received immediate treatment, while 70.7% experienced an intermediate delay and 7.20% endured a long delay prior to undergoing EC treatment.

**Table 1:** Demographic and clinical characteristics of the 12,911 esophageal cancer patients categorized by treatment delay from diagnosis to treatment.

Characteristics	All	Brief delay	Moderate delay	Long delay	P-value
**Total number**	12911	2852	9131	928	
**Age, 95%CI**	66.5 (66.3-66.7)	64.9 (64.5-65.4)	66.9 (66.7-67.1)	67.0 (66.3-67.7)	<0.001
**Survival months, 95%CI**	18.6 (18.1-19.0)	23.1 (21.3-25.0)	19.3 (18.7-19.9)	14.7 (13.8-15.7)	<0.001
Age group (years)					<0.001
≤44	378 (2.9)	115 (4.0)	237 (2.6)	26 (2.8)	
45-54	1609 (12.5)	443 (15.5)	1063 (11.6)	103 (11.1)	
55-64	3568 (27.6)	846 (29.7)	2478 (27.1)	244 (26.3)	
65-74	3873 (30.0)	786 (27.6)	2794 (30.6)	293 (31.6)	
≥75	3483 (27.0)	662 (23.2)	2559 (28.0)	262 (28.2)	
Sex					0.006
Male	10235 (79.3)	2318 (81.3)	7200 (78.9)	717 (77.3)	
Female	2676 (20.7)	534 (18.7)	1931 (21.1)	211 (22.7)	
Marital status					<0.001
Single	5360 (41.5)	1221 (42.8)	3690 (40.4)	449 (48.4)	
Couple	7551 (58.5)	1631 (57.2)	5441 (59.6)	479 (51.6)	
Household income					<0.001
Low level	1668 (12.9)	413 (14.5)	1142 (12.5)	113 (12.2)	
Middle level	5253 (40.7)	1167 (40.9)	3658 (40.1)	428 (46.1)	
High level	5990 (46.4)	1272 (44.6)	4331 (47.4)	387 (41.7)	
Living areas					0.003
Metropolitan areas	11127 (86.2)	2410 (84.5)	7896 (86.5)	821 (88.5)	
Nonmetropolitan areas	1784 (13.8)	442 (15.5)	1235 (13.5)	107 (11.5)	
Primary site					0.026
Upper third of esophagus	779 (6.0)	198 (6.9)	524 (5.7)	57 (6.1)	
Middle third of esophagus	2103 (16.3)	418 (14.7)	1525 (16.7)	160 (17.2)	
Lower third of esophagus	7604 (58.9)	1669 (58.5)	5401 (59.2)	534 (57.5)	
Other site	2425 (18.8)	567 (19.9)	1681 (18.4)	177 (19.1)	
Differentiation					<0.001
Highly differentiated	586 (4.5)	99 (3.5)	424 (4.6)	63 (6.8)	
Moderately differentiated	5133 (39.8)	1088 (38.1)	3646 (39.9)	399 (43.0)	
Poor differentiated	6954 (53.9)	1595 (55.9)	4902 (53.7)	457 (49.2)	
Undifferentiated	238 (1.8)	70 (2.5)	159 (1.7)	9 (1.0)	
Stage					<0.001
I	1358 (10.5)	248 (8.7)	945 (10.3)	165 (17.8)	
II	2139 (16.6)	315 (11.0)	1610 (17.6)	214 (23.1)	
III	2930 (22.7)	490 (17.2)	2212 (24.2)	228 (24.6)	
IV	6484 (50.2)	1799 (63.1)	4364 (47.8)	321 (34.6)	
Histology					0.011
Squamous cell carcinoma	4746 (36.8)	987 (34.6)	3380 (37.0)	379 (40.8)	
Adenocarcinoma	7119 (55.1)	1630 (57.2)	5015 (54.9)	474 (51.1)	
Other	1046 (8.1)	235 (8.2)	736 (8.1)	75 (8.1)	
Radiotherapy					<0.001
Yes	9680 (75.0)	1992 (69.8)	6944 (76.0)	744 (80.2)	
No	3231 (25.0)	860 (30.2)	2187 (24.0)	184 (19.8)	
Chemotherapy					<0.001
Yes	10857 (84.1)	2281 (80.0)	7853 (86.0)	723 (77.9)	
No	2054 (15.9)	571 (20.0)	1278 (14.0)	205 (22.1)	

95%CI, 95% confidential interval.

Among the EC patients, males constituted the largest group with a total of 79.3%, while females accounted for 20.7%. Brief treatment was received by only approximately one-fifth of male and female patients, with percentages of 22.6% and 19.9%, respectively. Delay in cancer treatment is consistently observed among single EC cancer patients (22.8%), residing in nonmetropolitan areas (24.8%) and having low household income (24.8%).

### Survival outcomes and univariate analysis

The OS rates at 1, 3, and 5 years for all patients were 37.8%(37.0%-38.7%), 12.6%(12.0%-13.2%), and 7.88%(7.42%-8.36%), respectively. The median OS time for the investigated cases of EC was 9.00 months (95% CI=8.78-9.21). The CSS rates at 1, 3, and 5 years for all patients were found to be 40.9%(40.0%-41.8%), 15.5%(14.9%-16.2%) and 11.1%(10.5%-11.7%), respectively. The median CSS time for the investigated EC cases was determined to be approximately 10.0 months (95% CI=9.76-10.2). The demographic and clinical characteristics were used to categorize the subgroup of OS and CSS rate, as well as their median survival time.

The OS rates of EC in male patients, patients aged ≥75, low-income patients, single patients, and residing in nonmetropolitan areas were significantly lower with a 5-year OS rate of only 6.77%, 7.23%, 1.30%, 7.43%, 6.78%, and 6.58%. The 5-year OS rates of patients with upper third esophageal tumors (15.5%) and patients diagnosed with squamous cell carcinoma (12.1%) exhibited superior outcomes compared to those observed in other patients. The 5-year OS rate of poor/undifferentiated patients was significantly lower compared to that of other patients. The patients in stage I exhibited the longest median survival time (14.0 months), whereas those in stage IV demonstrated the shortest median survival time (7.0 months). The CSS rate showed the similar findings. (**Table 2**; **Supplementary Table 1**)

**Table 2:** Overall survival rate and median survival time of the 12,911 esophageal cancer patients.

Characteristics	Overall survival rate (%, 95%CI)				
	1-year	3-year	5-year	Survival time (months, 95%CI)	P-value
**All patients**	37.8 (37.0-38.7)	12.6 (12.0-13.2)	7.88 (7.42-8.36)	9.00 (8.78-9.21)	
Sex					<0.001
Male	36.3 (35.4-37.2)	11.2 (10.6-11.8)	6.77 (6.29-7.28)	9.00 (8.76-9.23)	
Female	43.6 (41.8-45.6)	18.0 (16.6-19.5)	12.1 (10.9-13.4)	11.0 (10.4-11.5)	
Age group (years)					<0.001
≤44	34.8 (30.3-40.0)	9.61 (7.04-13.1)	6.87 (4.72-10.0)	9.00 (7.90-10.0)	
45-54	35.4 (33.1-37.8)	9.37 (8.04-10.9)	6.55 (5.44-7.89)	9.00 (8.46-9.53)	
55-64	36.8 (35.2-38.4)	11.9 (10.9-13.0)	7.52 (6.70-8.44)	9.00 (8.63-9.37)	
65-74	40.9 (39.4-42.5)	14.9 (13.8-16.1)	9.42 (8.54-10.4)	10.0 (9.58-10.4)	
≥75	36.9 (35.3-38.5)	12.5 (11.5-13.7)	7.23 (6.42-8.15)	8.00 (7.56-8.43)	
Marital status					<0.001
Single	35.4 (34.1-36.7)	11.4 (10.6-12.3)	6.78 (6.13-7.49)	9.00 (8.68-9.31)	
Couple	39.5 (38.4-40.6)	13.4 (12.6-14.2)	8.65 (8.04-9.31)	10.0 (9.72-10.2)	
Household income					<0.001
Low level	34.7 (32.5-37.0)	11.7 (10.2-13.3)	7.43 (6.27-8.81)	8.00 (7.42-8.57)	
Middle level	36.6 (35.3-37.9)	12.4 (11.5-13.3)	7.73 (7.03-8.49)	9.00 (8.67-9.32)	
High level	39.8 (38.5-41.0)	13.0 (12.2-13.9)	8.13 (7.46-8.85)	10.0 (9.68-10.3)	
Living areas					0.001
Metropolitan areas	38.3 (37.4-39.2)	12.9 (12.3-13.5)	8.08 (7.59-8.61)	9.00 (8.76-9.23)	
Nonmetropolitan areas	34.8 (32.6-37.0)	10.8 (9.46-12.3)	6.58 (5.52-7.85)	9.00 (8.45-9.54)	
Primary site					<0.001
Upper third of esophagus	48.4 (45.0-52.0)	21.5 (18.8-24.6)	15.5 (13.1-18.3)	12.0 (10.8-13.1)	
Middle third of esophagus	39.9 (37.8-42.0)	16.6 (15.1-18.3)	10.1 (8.92-11.5)	10.0 (9.47-10.5)	
Lower third of esophagus	36.7 (35.6-37.8)	10.1 (9.52-10.8)	6.33 (5.80-6.91)	9.00 (8.73-9.26)	
Other site	36.1 (34.2-38.1)	14.0 (12.7-15.4)	8.31 (7.28-9.49)	8.00 (7.52-8.48)	
Diferentiation					<0.001
Highly diferentiated	50.0 (46.2-54.3)	21.6 (18.5-25.2)	12.9 (10.5-16.0)	13.0 (11.7-14.2)	
Moderately diferentiated	42.4 (41.0-43.7)	15.1 (14.1-16.1)	9.27 (8.50-10.1)	10.0 (9.64-10.3)	
Poor diferentiated	33.7 (32.6-34.8)	10.1 (9.44-10.8)	7.56 (4.85-11.7)	8.00 (7.74-8.25)	
Undiferentiated	29.4 (24.1-35.8)	10.0 (6.90-14.7)	6.43 (5.87-7.03)	8.00 (6.74-9.25)	
Stage					<0.001
I	53.2 (50.7-56.0)	24.7 (22.5-27.1)	15.0 (13.2-17.0)	14.0 (12.8-15.1)	
II	53.0 (50.9-55.2)	22.7 (21.0-24.6)	14.5 (13.1-16.1)	14.0 (13.1-14.8)	
III	45.7 (43.9-47.5)	17.3 (16.0-18.8)	11.2 (10.1-12.4)	11.0 (10.4-11.5)	
IV	26.0 (24.9-27.1)	4.57 (4.09-5.12)	2.61 (2.25-3.04)	7.00 (6.77-7.22)	
Histology					<0.001
Squamous cell carcinoma	41.2 (39.8-42.6)	18.6 (17.5-19.7)	12.1 (11.2-13.1)	10.0 (9.62-10.3)	
Adenocarcinoma	36.6 (35.5-37.7)	9.31 (8.66-10.0)	5.38 (4.88-5.94)	9.00 (8.72-9.27)	
Other	31.0 (28.3-33.9)	8.16 (6.65-10.0)	5.11 (4.65-5.90)	7.00 (6.35-7.64)	
Radiotherapy					<0.001
Yes	40.8 (39.8-41.7)	15.2 (14.5-15.9)	9.51 (8.94-10.1)	10.0 (9.72-10.2)	
No	26.0 (24.9-27.1)	4.57 (4.09-5.12)	2.61 (2.25-3.04)	8.00 (7.66-8.33)	
Chemotherapy					<0.001
Yes	41.4 (40.5-42.3)	14.0 (1.34-14.7)	8.86 (8.34-9.41)	10.0 (9.71-10.2)	
No	18.8 (17.2-20.6)	4.94 (4.08-5.98)	2.67 (2.05-3.48)	4.00 (3.67-4.32)	
Time from diagnosis to treatment					<0.001
Brief delay	50.2 (47.0-53.5)	16.9 (14.7-19.5)	10.0 (8.25-12.1)	13.0 (12.1-13.8)	
Moderate delay	39.5 (38.5-40.5)	13.4 (12.7-14.1)	8.31 (7.75-8.89)	10.0 (9.74-10.2)	
Long delay	28.4 (26.8-30.1)	8.63 (7.65-9.73)	5.80 (4.99-6.73)	7.00 (6.61-7.38)	

95%CI, 95% confidential interval.

**Figure 2:**
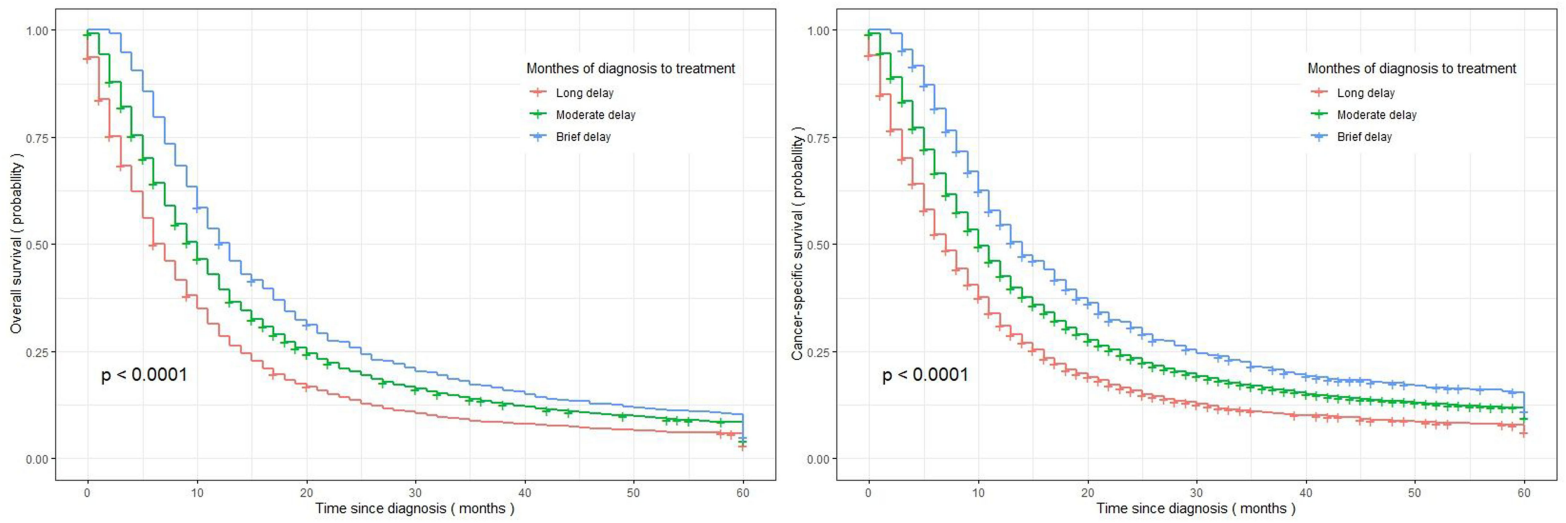
Kaplan-Meier curves illustrating the overall survival and cancer-specific survival of the investigated EC patients, stratified by treatment delay (left panel: overall survival; right panel: cancer-specific survival).

Patients with EC who experienced a long delay between diagnosis and treatment exhibited the lowest OS and CSS, with median survival times of 7.88 months (95% CI=7.42-8.36) and 10.0 months (95% CI=9.76-10.2), respectively. The figures illustrate in **Figure 2** that patients who experienced a long treatment delay exhibited the lowest survival rates within 60 months, whereas those who did not encounter any delay had the highest survival rates.

The univariate and multivariate analysis presented in **Table 3**; **Supplementary Table 2** also provide corroborative evidence for these findings. Following adjustment for relevant covariates, patients with EC who experienced an intermediate delay in treatment exhibited a significantly elevated risk of all-cause mortality (HR=1.209, 95%CI=1.128-1.296) and cancer-specific mortality (HR=1.259, 95%CI=1.168-1.358) compared to the group receiving immediate treatment. Similar results were observed in the cohort with long delay in treatment, where EC patients demonstrated a significantly higher risk of all-cause mortality (HR=1.574, 95%CI=1.459-1.698) and cancer-specific mortality (HR=1.693, 95%CI=1.560-1.838).

**Table 3:** Univariate and multivariate analyses demonstrating the association between demographic factors, clinical characteristics, and overall survival in patients with esophageal cancer.

Characteristics	Univariate		Multivariate	
	HR (95%CI)	p-value	HR (95%CI)	p-value
Sex				
Male	Reference		Reference	
Female	0.841 (0.802-0.881)	<0.001	**0.809 (0.774-0.846)**	<0.001
**Age group (years)**		<0.001		<0.001
≤44	Reference		Reference	
45-54	1.014 (0.904-1.138)	0.809	1.060 (0.944-1.190)	0.323
55-64	0.976 (0.875-1.088)	0.658	1.068 (0.957-1.192)	0.241
65-74	0.911 (0.817-1.015)	0.091	1.062 (0.952-1.186)	0.281
≥75	1.045 (0.937-1.166)	0.427	**1.261 (1.128-1.410)**	<0.001
Marital status				
Single	Reference		Reference	
Couple	0.852 (0.821-0.885)	<0.001	**0.902 (0.871-0.935)**	<0.001
**Household income**		<0.001		<0.001
Low level	Reference		Reference	
Middle level	0.948 (0.890-1.010)	0.101	0.967 (0.914-1.023)	0.242
High level	0.882 (0.824-0.943)	<0.001	**0.911 (0.862-0.963)**	0.001
Living areas				
Metropolitan areas	Reference		Reference	
Nonmetropolitan areas	1.022 (0.962-1.085)	0.487	**1.087 (1.033-1.144)**	0.001
**Primary site**		0.007		<0.001
Upper third of esophagus	Reference		Reference	
Middle third of esophagus	1.108 (1.017-1.208)	0.019	**1.194 (1.096-1.301)**	<0.001
Lower third of esophagus	1.084 (0.997-1.179)	0.058	**1.365 (1.263-1.474)**	<0.001
Other site	1.149 (1.054-1.251)	0.002	**1.317 (1.211-1.432)**	<0.001
**Differentiation**		<0.001		<0.001
Highly diferentiated	Reference		Reference	
Moderately diferentiated	1.149 (1.051-1.257)	0.002	**1.182 (1.082-1.292)**	<0.001
Poor diferentiated	1.328 (1.216-1.451)	<0.001	**1.440 (1.319-1.572)**	<0.001
Undiferentiated	1.230 (1.052-1.439)	0.010	**1.472 (1.261-1.719)**	<0.001
**Stage**		<0.001		
I	Reference		Reference	
II	1.103 (1.027-1.184)	0.007	0.991 (0.924-1.064)	0.807
III	1.330 (1.243-1.424)	<0.001	**1.173 (1.097-1.253)**	0.001
IV	2.075 (1.944-2.215)	<0.001	**1.978 (1.861-2.102)**	0.001
**Histology**		<0.001		<0.001
Squamous cell carcinoma	Reference		Reference	
Adenocarcinoma	1.061 (1.010-1.115)	0.019	**1.235 (1.189-1.283)**	<0.001
Other	1.232 (1.144-1.328)	<0.001	**1.403 (1.310-1.502)**	<0.001
Radiotherapy				
Yes	Reference		Reference	
No	1.163 (1.111-1.218)	<0.001	**1.408 (1.351-1.466)**	<0.001
Chemotherapy				
Yes	Reference		Reference	
No	2.093 (1.989-2.203)	<0.001	**1.932 (1.841-2.026)**	<0.001
**Time from diagnosis to treatment**		<0.001		<0.001
Brief delay	Reference		Reference	
Moderate delay	1.246 (1.161-1.336)	<0.001	**1.209 (1.128-1.296)**	<0.001
Long delay	1.503 (1.392-1.623)	<0.001	**1.574 (1.459-1.698)**	<0.001

HR, hazard ratio; 95%CI, 95% confidential interval. Signifcant values are in [bold].

### Multivariable analysis

The Cox analyses further validated that sex, age, marital status, household income, living areas, primary site, differentiation, stage, histology, radiotherapy and chemotherapy exhibited significant associations with an elevated risk of both all-cause and cancer-specific mortality.

### Dose-response analysis

**Figure 3:**
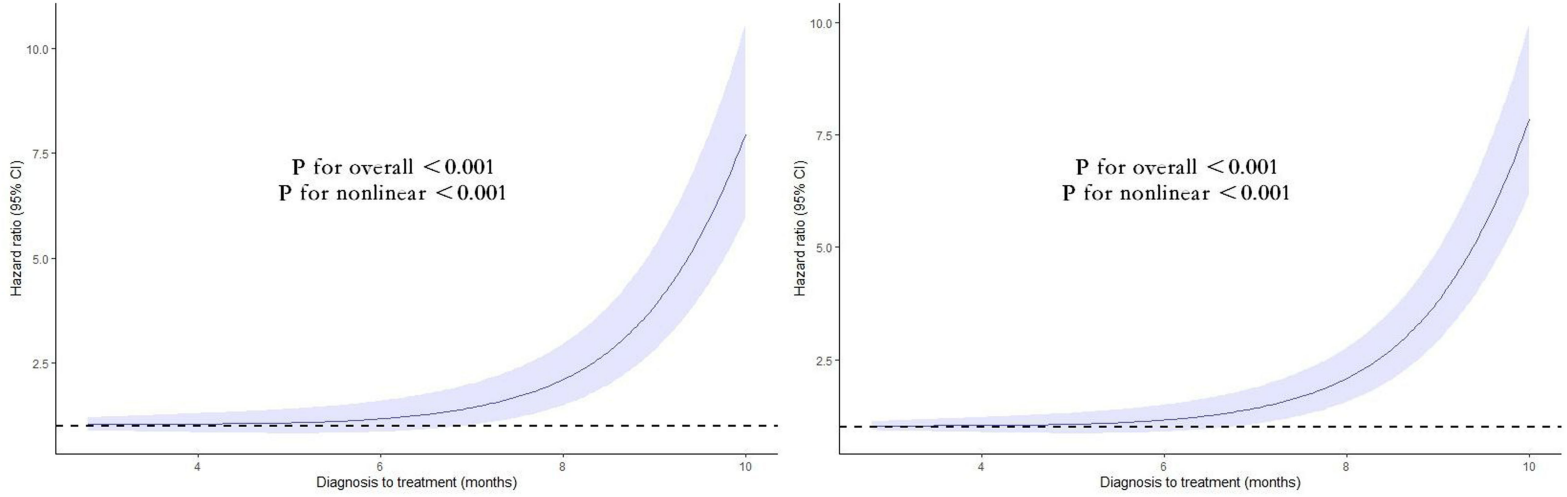
Nonlinear association between the duration from diagnosis to treatment and the risk of mortality, both all-cause (left) and cause-specific (right). Associations were assessed using multivariable Cox regression models with restricted cubic splines.

A non-linear relationship between the duration from diagnosis to treatment and the risk of mortality, both all-cause and cause-specific, is depicted in **Figure 3**. A statistically significant J-shaped correlation was observed between treatment delay and both overall mortality as well as mortality related to specific causes. The findings suggest that there is a positive correlation between the duration of treatment delay and the patient’s risk of mortality, both from all-cause and cancer-specific causes.

### Subgroup analysis

We conducted subgroup analysis stratified by the demographic and clinical covariates. The observed trends suggest that most subgroups exhibit similar effects on treatment delay and OS, with patients experiencing long delays being at a higher risk of mortality. The adverse impact of prolong delay of treatment on the survival of patients with EC was consistently observed across various subgroups. (**Table 4**)

**Table 4:** Subgroup analyses for the association of the association between demographic factors, clinical characteristics, and overall survival in patients with esophageal cancer.

Characteristics	Brief delay	Moderate delay	Long delay	P-value
**Total number**	2852	9131	928	
Sex				
Male	Reference	**1.257 (1.161-1.360)**	**1.476 (1.354-1.610)**	<0.001
Female	Reference	**1.235 (1.063-1.435)**	**1.609 (1.360-1.905)**	<0.001
Age group (years)				
≤44	Reference	1.420 (0.911-2.211)	1.376 (0.855-2.215)	0.300
45-54	Reference	**1.442 (1.157-1.797)**	**1.714 (1.360-2.160)**	<0.001
55-64	Reference	1.110 (0.968-1.272)	**1.296 (1.117-1.503)**	<0.001
65-74	Reference	**1.286 (1.133-1.459)**	**1.600 (1.390-1.842)**	<0.001
≥75	Reference	**1.288 (1.130-1.468)**	**1.679 (1.449-1.946)**	<0.001
Primary site				
Upper third of esophagus	Reference	**1.492 (1.114-1.997)**	**1.857 (1.358-2.538)**	<0.001
Middle third of esophagus	Reference	**1.272 (1.074-1.508)**	**1.660 (1.372-2.009)**	<0.001
Lower third of esophagus	Reference	**1.250 (1.140-1.370)**	**1.439 (1.301-1.592)**	<0.001
Other site	Reference	**1.176 (1.000-1.383)**	**1.466 (1.229-1.749)**	<0.001
Diferentiation				
Highly diferentiated	Reference	1.154 (0.866-1.537)	**1.507 (1.073-2.117)**	0.035
Moderately diferentiated	Reference	**1.315 (1.180-1.465)**	**1.514 (1.343-1.707)**	<0.001
Poor diferentiated	Reference	**1.190 (1.078-1.314)**	**1.468 (1.318-1.635)**	<0.001
Undiferentiated	Reference	1.374 (0.67-2.818)	1.624 (0.773-3.416)	0.351
Stage				
I	Reference	1.082 (0.910-1.286)	**1.173 (1.055-1.442)**	0.035
II	Reference	**1.234 (1.062-1.434)**	**1.354 (1.127-1.626)**	0.005
III	Reference	1.071 (0.929-1.234)	**1.203 (1.022-1.418)**	0.037
IV	Reference	**1.476 (1.314-1.658)**	**1.837 (1.626-2.075)**	<0.001
Radiotherapy				
Yes	Reference	**1.255 (1.160-1.358)**	**1.527 (1.398-1.667)**	<0.001
No	Reference	**1.293 (1.106-1.512)**	**1.490 (1.263-1.757)**	<0.001
Chemotherapy				
Yes	Reference	**1.164 (1.076-1.261)**	**1.321 (1.211-1.441)**	<0.001
No	Reference	**1.444 (1.244-1.678)**	**2.223 (1.887-2.620)**	<0.001
Marital status				
Single	Reference	**1.225 (1.107-1.356)**	**1.594 (1.425-1.783)**	<0.001
Couple	Reference	**1.274 (1.156-1.404)**	**1.446 (1.300-1.609)**	<0.001
Household income				
Low level	Reference	1.082 (0.888-1.319)	**1.331 (1.075-1.647)**	0.001
Middle level	Reference	**1.298 (1.170-1.442)**	**1.568 (1.397-1.760)**	<0.001
High level	Reference	**1.245 (1.118-1.388)**	**1.476 (1.311-1.662)**	<0.001
Living areas				
Metropolitan areas	Reference	**1.257 (1.167-1.355)**	**1.504 (1.385-1.633)**	<0.001
Nonmetropolitan areas	Reference	1.193 (0.971-1.465)	**1.482 (1.190-1.846)**	<0.001
Histology				
Squamous cell carcinoma	Reference	**1.377 (1.231-1.540)**	**1.685 (1.487-1.908)**	<0.001
Adenocarcinoma	Reference	**1.181 (1.072-1.302)**	**1.373 (1.235-1.526)**	<0.001
Other	Reference	1.212 (0.943-1.594)	**1.624 (1.233-2.272)**	<0.001

95%CI, 95% confidential interval. Signifcant values are in [bold].

The detrimental impact of long treatment delay on the CSS was consistently observed across various subgroups. (**Supplementary Table 3**)

### Assessment of Cox proportional hazard regression models

**Figure 4:**
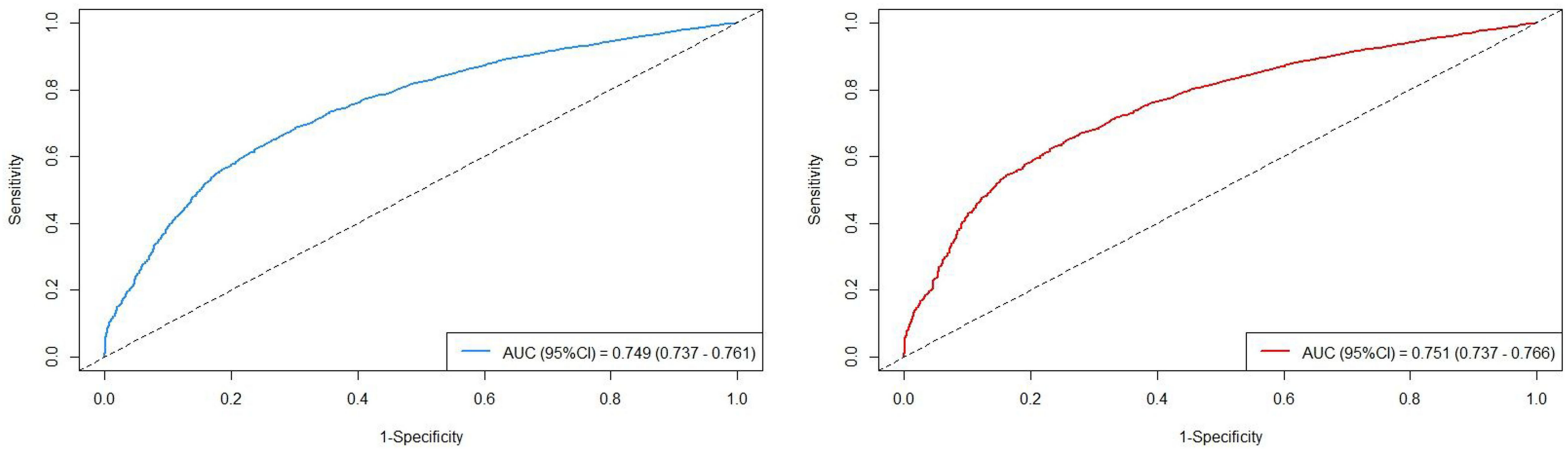
The AUC values in the cohort, stratified by survival outcomes (left: overall survival; right: cancer-specific survival).

The AUC values of the two models with different outcomes are presented in **Figure 4**. The AUC values in the cohort were observed to be high: AUC_OS_ =0.749 (95%CI=0.737-0.761) and AUC_CSS_ =0.751 (95%CI=0.737-0.766). All model assessment results confirmed that our Cox models fit well.

## Discussion

The aim of the present study is to investigate the impact of delayed endoscopic treatment on overall survival (OS) and cancer-specific survival (CSS) in nonsurgical esophageal cancer patients, while controlling for individual and clinical characteristics, utilizing data from the period spanning 2000 to 2020. Patients who experienced a long delay (≥3 months) in EC treatment exhibited 1-, 3-, and 5-year OS rates of 28.4%, 8.63%, and 5.80% respectively, with corresponding CSS rates of 31.0%, 10.7%, and 7.79%. Notably, an independent association was observed between long delay in EC treatment and significantly higher all-cause mortality as well as mortality specifically attributed to cancer-related causes among patients with EC. Moreover, a statistically significant J-shaped correlation was identified between treatment delay and both overall mortality as well as cause-specific mortality outcomes. These findings underscored the importance of investigating the impact of treatment delays on long-term survival outcomes in patients with EC.

The accumulation of substantial evidence indicates that a delay in the initiation of cancer treatment can lead to unfavorable outcomes (7, 24, 25). A comprehensive meta-analysis revealed that even a mere four-week delay in cancer treatment is associated with an increased risk of mortality across various types of cancer, including both surgical and nonsurgical treatments. The consistent findings consistently indicate a mortality risk ranging from 1.06 to 1.08 for every four-week delay in nonsurgical procedures (7). Similarly, a large cohort study indicated that there is a consistent association between delayed initiation of treatment and increased 5-year and 10-year predicted mortality rates across various cancers, including nonmetastatic breast, prostate, non-small cell lung, and colon cancers. This study also provided evidence suggesting that shorter time-to-treatment initiation is linked to reduced mortality rates for all examined cancer types, implying an indirect relationship between treatment deferral and mortality (24). The findings of another extensive cohort study suggested a significant prolongation in the time to treatment initiation, which is associated with an absolute increase in mortality risk ranging from 1.2% to 3.2% per week in curative settings such as early-stage breast, lung, renal, and pancreatic cancers (25). Recently conducted multi-cancer analyses revealed that, upon adjusting for confounding factors, a prolonged duration from diagnosis to treatment initiation (<6 months) was associated with adverse effects on the survival outcomes of patients diagnosed with early-stage female cancers, including non-small cell lung cancer, breast cancer, thyroid cancer, colorectal cancer, and cervical cancer (18). However, previous studies have primarily focused on the impact of treatment delays on survival outcomes in other prevalent cancers. The occurrence of treatment delay in anti-tumor therapy is more likely to be observed among both non-surgical candidates with early-stage cancer and patients who are not recommended for surgery due to advanced-stage cancer (26–28).

The continuous updates from systematic reviews and meta-analyses have consistently demonstrated the detrimental impact of prolonged wait times between diagnosis and treatment on clinical outcomes among patients with various types of cancer (7, 15, 29). Additionally, this period preceding treatment can be distressing for patients, as they grapple with the emotional and psychological impact of their diagnosis. This waiting period, albeit necessary, can adversely affect their quality of life, causing stress, anxiety, and depression. Delays in the treatment of patients with localized cancers following their initial diagnoses have been shown to increase the likelihood of disease progression to locally advanced or even metastatic stages. The prognosis of EC is strongly correlated with the stages as per the tumor-node-metastasis (TNM) staging system established by the American Joint Committee on Cancer (AJCC) (30). In Stage I, surgery treatment intervention yields a 5-year survival rate ranging from 50% to 80%. For Stages II and III, the corresponding 5-year survival rates are approximately 30-40% and 10-15%, respectively. Patients diagnosed with metastatic disease (Stage IV) who receive palliative therapy have a median survival of less than one year (31). However, the 5-year OS and CSS rates for non-surgical EC patients in our study were only 5.80% (4.99%-6.73%) and 7.79% (6.79%-8.94%), respectively. Therefore, early detection and timely treatment in Stages I or II offer a significant potential for cure among EC patients. Interestingly, we observed a significant increase in the risk of both all-cause and cancer-specific mortality among patients with all stages of EC who experienced long treatment delays compared to those without any delay in treatment. The findings implied that a delay in treatment for EC could potentially lead to increased the risk of mortality among non-surgical EC patients at any stage of EC.

In addition to the clinical characteristics, our studies also indicated that socio-demographic factors may influence the time interval between diagnosis and treatment. It is important to note that disparities in demographic factors may have an impact on the duration of cancer treatment delay as revealed in our study. These factors included sex, age, marital status, household income, and living areas. Our findings found that male patients, those in higher age groups, single individuals, those with lower household incomes, and those residing in metropolitan areas were more likely to experience long delays in non-surgical esophageal cancer treatment. Furthermore, previous studies have also suggested that ethnicity is a significant socio-demographic factor. Adams et al. found that American Indian and Alaska Natives tend to initiate cancer therapy at a later stage compared to non-Hispanic Whites (32).

The collective findings indicate a pressing necessity to reassess the organization of our cancer services. The prevailing paradigm has focused on improving access to new treatments for better outcomes, but at a systemic level, prioritizing efforts to reduce the time from cancer diagnosis to treatment initiation from weeks to days could lead to gains in survival. While acknowledging that delays in treatment are multifactorial and patients should not begin treatment until medically cleared and all appropriate evaluations have been completed, these data strongly support minimizing system-level delays. For instance, national quality indicators regarding cancer waiting times from diagnosis to treatment are widely utilized across various healthcare systems. In the UK NHS, current targets for initiating primary definitive treatment have been set at 31 days from the decision-to-treat date; this does not include the lag between receiving a diagnosis and having a surgical or radiation oncology consultation for treatment (33). In the Netherlands, the current standard of care advocates for a reduction in the interval between diagnosis and treatment to a maximum of 5 weeks. This is to ensure that patients receive prompt and effective care, which is crucial in improving their chances of recovery. To enforce this standard, all Dutch hospitals are mandated to publish their waiting times on a monthly basis, submitting them to the Dutch Healthcare authority for monitoring and evaluation purposes (34, 35). Many effective strategies have been implemented to reduce delays in treatment, such as enhancing the capacity of specialist workforce through training initiatives and addressing these challenges through technological advancements. The standardization of automated treatment contouring and planning has significantly decreased radiotherapy preparation time from days to mere hours (36). The establishment of satellite centers can also enhance patient treatment capacity, along with re-configuring existing infrastructure to accommodate high-volume super specialized services or adopting single entry models and team-based care approaches (37).

The current study has certain limitations that require attention. Firstly, it is a retrospective observational study with potential bias in participant selection, uneven baseline characteristics, and other factors that may confound the results. Secondly, curent database lacks information on patient attributes such as lifestyle choices, educational background, insurance status, Charlson-Deyo comorbidity index score, mental health status or medical knowledge which could have influenced their prognosis. Additionally, the available database does not provide detailed records regarding reasons for treatment delays which are crucial information for further investigation into this important topic and reducing cancer progression due to delayed treatment.

## Conclusion

The prolonged delay in initiating treatment significantly impacts the OS and CSS outcomes for patients with non-surgical EC cases. Timely administration of therapy has the potential to improve the survival outcomes of individuals diagnosed with EC who are not eligible for surgical intervention, including those in advanced stages where surgical options may be not recommended or deemed inappropriate due to disease severity.

## Data availability statement

The raw data supporting the conclusions of this article will be made available by the authors, without undue reservation.

## Ethics statement

The studies involving humans were approved by Ethical approval in this study was obtained from the Institutional Review Board (IRB) of Zibo Central Hospital (IRB2022-00001032). The studies were conducted in accordance with the local legislation and institutional requirements. Written informed consent for participation in this study was provided by the participants’ legal guardians/next of kin.

## Author contributions

YS: Conceptualization, Formal analysis, Funding acquisition, Investigation, Methodology, Project administration, Software, Supervision, Writing – original draft, Writing – review & editing. PZ: Formal analysis, Funding acquisition, Writing – original draft, Writing – review & editing. DZ: Conceptualization, Data curation, Investigation, Methodology, Project administration, Resources, Supervision, Validation, Visualization, Writing – original draft, Writing – review & editing.
