# Supplementary material for: Impact of treatment delays on future survival outcomes in non-surgical patients with esophageal cancer in Shandong Province, China
Source: Front Oncol. 2024 Jul 19;14:1445267. doi: 10.3389/fonc.2024.1445267 (PMC11294235; doi:10.3389/fonc.2024.1445267)
Supplement: Supplementary file 1 [file Table_1.docx]

**Supplementary Table 1.** Cancer-specific survival rate and median survival time of the 12911 esophageal cancer patients.

| **Characteristics** | **Overall survival rate (%, 95%CI)** | | |  |  |
| --- | --- | --- | --- | --- | --- |
|  | **1-year** | **3-year** | **5-year** | **Survival time**  **(months, 95%CI)** | **P-value** |
| **All patients** | 40.9(40.0-41.8) | 15.5(14.9-16.2) | 11.1(10.5-11.7) | 10.0(9.76-10.2) |  |
| **Sex** |  |  |  |  | ＜0.001 |
| Male | 39.3(38.4-40.3) | 13.9(13.2-14.6) | 9.72(9.11-10.3) | 10.0(9.75-10.2) |  |
| Female | 46.8(44.8-48.7) | 21.7(20.1-23.4) | 16.6(15.1-18.2) | 11.0(10.3-11.6) |  |
| **Age group (years)** |  |  |  |  | ＜0.001 |
| ≤44 | 35.7(31.1-40.9） | 11.1(8.24-14.9) | 7.93(5.51-11.4) | 9.00(8.02-9.97) |  |
| 45-54 | 38.0(35.6-40.5) | 11.4(9.88-13.1) | 8.53(7.17-10.1) | 9.00(8.41-9.58) |  |
| 55-64 | 39.3(37.7-40.9) | 13.9(12.8-15.2) | 9.96(8.96-11.0) | 10.0(9.59-10.4) |  |
| 65-74 | 44.0(42.4-45.6) | 18.1(16.9-19.4) | 13.0(11.9-14.2) | 11.0(10.5-11.4) |  |
| ≥75 | 41.1(39.4-42.8) | 1.68(15.5-18.2) | 11.9(10.7-13.2) | 10.0(9.50-10.4) |  |
| **Marital status** |  |  |  |  | ＜0.001 |
| Single | 38.8(37.5-40.2) | 14.8(13.8-15.8) | 10.4(9.55-11.3) | 9.00(8.64-9.35) |  |
| Couple | 42.3(41.2-43.5) | 16.1(15.2-17.0) | 11.6(10.9-12.4) | 10.0(9.68-10.3) |  |
| **Household income** |  |  |  |  | ＜0.001 |
| Low level | 37.9(35.6-40.4) | 15.0(13.3-16.9) | 10.8(9.30-12.6) | 9.00(8.38-9.61) |  |
| Middle level | 39.6(38.3-41.0) | 14.9(13.9-16.0) | 11.0(10.1-12.0) | 9.00(8.63-9.36) |  |
| High level | 42.8(41.6-44.1) | 16.2(15.3-17.2) | 11.3(10.5-12.2) | 11.0(10.6-11.3) |  |
| **Living areas** |  |  |  |  | ＜0.001 |
| Metropolitan areas | 41.3(40.4-42.3) | 15.9(15.2-16.6) | 11.4(10.8-12.1) | 10.0(9.74-10.2) |  |
| Nonmetropolitan areas | 38.0(35.8-40.4) | 13.4(11.8-15.2) | 9.18(7.82-10.7) | 9.00(8.42-9.57) |  |
| **Primary site** |  |  |  |  | ＜0.001 |
| Upper third of esophagus | 51.6(48.2-55.4) | 25.9(22.8-29.4) | 21.3(18.4-24.7) | 13.0(11.5-14.4) |  |
| Middle third of esophagus | 43.6(41.5-45.9) | 20.7(19.0-22.7) | 14.8(13.2-16.6) | 11.0(10.3-11.6) |  |
| Lower third of esophagus | 39.5(38.4-40.6) | 12.5(11.7-13.3) | 8.85(8.19-9.58) | 10.0(9.71-10.2) |  |
| Other site | 39.5(37.5-41.5) | 17.4(15.9-19.1) | 12.0(10.7-13.6) | 9.00(8.39-9.60) |  |
| **Diferentiation** |  |  |  |  | ＜0.001 |
| Highly diferentiated | 54.4(50.5-58.7) | 26.4(22.9-30.4) | 19.5(16.3-23.3) | 14.0(12.2-15.7) |  |
| Moderately diferentiated | 45.7(44.4-47.2) | 18.4(17.3-19.5) | 12.8(11.8-13.8) | 11.0(10.5-11.4) |  |
| Poor diferentiated | 36.5(35.4-37.7) | 12.6(11.8-13.5) | 9.32(8.60-10.1) | 9.00(8.70-9.29) |  |
| Undiferentiated | 30.5(25.2-37.1) | 11.6(8.14-16.7) | 9.17(6.04-13.9) | 8.00(6.76-9.23) |  |
| **Stage** |  |  |  |  | ＜0.001 |
| Ⅰ | 59.7(57.0-62.4) | 33.1(30.5-35.9) | 24.2(21.7-26.9) | 18.0(16.3-19.6) |  |
| Ⅱ | 57.4(55.3-59.6) | 29.0(27.1-31.2) | 21.6(19.8-23.6) | 16.0(14.8-17.1) |  |
| Ⅲ | 49.1(47.3-51.0) | 20.6(19.1-22.3) | 15.3(13.9-16.8) | 12.0(11.3-12.6) |  |
| Ⅳ | 27.9(26.8-29.0) | 5.41(4.86-6.03) | 3.36(2.92-3.87) | 7.00(6.77-7.22) |  |
| **Histology** |  |  |  |  | ＜0.001 |
| Squamous cell carcinoma | 44.8(43.3-46.3) | 23.2(21.9-24.5) | 17.5(16.3-18.8) | 11.0(10.5-11.4) |  |
| Adenocarcinoma | 39.4(38.3-40.6) | 11.3(10.5-12.1) | 7.56(6.92-8.26) | 10.0(9.70-10.2) |  |
| Other | 33.5(30.7-36.5) | 10.3(8.57-12.5) | 7.46(5.91-9.42) | 8.00(7.26-8.73) |  |
| **Radiotherapy** |  |  |  |  | ＜0.001 |
| Yes | 44.4(43.4-45.4) | 17.0(16.3-17.8) | 12.3(11.7-13.0) | 11.0(10.7-11.2) |  |
| No | 21.9(20.1-23.9) | 7.48(6.31-8.87) | 4.58(3.62-5.78) | 8.00(7.65-8.34) |  |
| **Chemotherapy** |  |  |  |  | ＜0.001 |
| Yes | 44.1(43.1-45.1) | 18.7(17.9-19.6) | 13.6(12.8-14.3) | 11.0(10.7-11.2) |  |
| No | 31.2(29.6-32.9) | 6.08(5.26-7.03) | 3.90(3.23-4.71) | 4.00(3.64-4.35) |  |
| **Time from diagnosis to treatment** |  |  |  |  | ＜0.001 |
| Brief delay | 54.5(51.3-57.9) | 21.2(18.5-24.2) | 15.1(12.8-18.0) | 14.0(12.8-15.1) |  |
| Moderate delay | 42.6(41.5-43.6) | 16.5(15.7-17.3) | 11.8(11.1-12.5) | 10.0(9.71-10.2) |  |
| Long delay | 31.0(29.3-32.8) | 10.7(9.56-11.9) | 7.79(6.79-8.94) | 7.00(6.59-7.40) |  |

95%CI: 95% confidential interval

**Supplementary Table 2.** Univariate and multivariate analyses demonstrating the association between demographic factors, clinical characteristics, and cancer-specific survival in patients with esophageal cancer.

| **Characteristics** | **Univariate** |  | **Multivariate** |  |
| --- | --- | --- | --- | --- |
|  | **HR(95%CI)** | **p-value** | **HR(95%CI)** | **p-value** |
| **Sex** |  |  |  |  |
| Male | Reference |  | Reference |  |
| Female | 0.861(0.819-0.905) | ＜0.001 | **0.802(0.765-0.841)** | ＜0.001 |
| **Age group (years)** |  | ＜0.001 |  | ＜0.001 |
| ≤44 | Reference |  | Reference |  |
| 45-54 | 1.035(0.906-1.172) | 0.437 | 1.032(0.916-1.162) | 0.606 |
| 55-64 | 1.045(0.905-1.161) | 0.504 | 1.035(0.925-1.158) | 0.547 |
| 65-74 | 1.009(0.911-1.134) | 0133 | 1.010(0.903-1.131) | 0.856 |
| ≥75 | 1.152(1.020-1.292) | ＜0.001 | **1.149(1.024-1.289)** | 0.018 |
| **Marital status** |  |  |  |  |
| Single | Reference |  | Reference |  |
| Couple | 0.869(0.835-0.904) | ＜0.001 | **0.922(0.888-0.958)** | ＜0.001 |
| **Household income** |  | ＜0.001 |  | 0.001 |
| Low level | Reference |  | Reference |  |
| Middle level | 0.958(0.896-1.026) | 0.219 | 0.982(0.925-1.043) | 0.561 |
| High level | 0.885(0.824-0.951) | 0.001 | **0.921(0.868-0.977)** | 0.007 |
| **Living areas** |  |  |  | 0.004 |
| Metropolitan areas | Reference |  | Reference |  |
| Nonmetropolitan areas | 1.016(0.953-1.083) | 0.63 | **1.083(1.026-1.144)** | ＜0.001 |
| **Primary site** |  | 0.016 |  | ＜0.001 |
| Upper third of esophagus | Reference |  | Reference |  |
| Middle third of esophagus | 1.098(0.999-1.205) | 0.051 | **1.197(1.091-1.314)** | ＜0.001 |
| Lower third of esophagus | 1.078(0.984-1.180) | 0.106 | **1.418(1.304-1.542)** | ＜0.001 |
| Other site | 1.146(1.044-1.257) | 0.004 | **1.357(1.238-1.486)** | ＜0.001 |
| **Diferentiation** |  | ＜0.001 |  | ＜0.001 |
| Highly diferentiated | Reference |  | Reference |  |
| Moderately diferentiated | 1.183(1.073-1.304) | 0.001 | **1.231(1.117-1.357)** | ＜0.001 |
| Poor diferentiated | 1.372(1.245-1.511) | ＜0.001 | **1.521(1.382-1.674)** | ＜0.001 |
| Undiferentiated | 1.314(1.113-1.551) | 0.001 | **1.622(1.377-1.911)** | ＜0.001 |
| **Stage** |  | ＜0.001 |  | ＜0.001 |
| Ⅰ | Reference |  | Reference |  |
| Ⅱ | 1.206(1.112-1.309) | ＜0.001 | 1.083(0.999-1.174) | 0.052 |
| Ⅲ | 1.548(1.433-1.672) | ＜0.001 | **1.368(1.269-1.476)** | ＜0.001 |
| Ⅳ | 2.513(2.334-2.705) | ＜0.001 | **2.442(2.278-2.618)** | ＜0.001 |
| **Histology** |  | ＜0.001 |  | ＜0.001 |
| Squamous cell carcinoma | Reference |  | Reference |  |
| Adenocarcinoma | 1.086(1.030-1.145) | 0.002 | **1.289(1.237-1.342)** | ＜0.001 |
| Other | 1.273(1.176-1.377) | ＜0.001 | **1.472(1.369-1.582)** | ＜0.001 |
| **Radiotherapy** |  |  |  |  |
| Yes | Reference |  | Reference |  |
| No | 1.171(1.116-1.228) | ＜0.001 | **1.482(1.42-1.546)** | ＜0.001 |
| **Chemotherapy** |  |  |  |  |
| Yes | Reference |  | Reference |  |
| No | 2.183(2.068-2.304) | ＜0.001 | **1.921(1.826-2.021)** | ＜0.001 |
| **Time from diagnosis to treatment** |  | ＜0.001 |  | ＜0.001 |
| Brief delay | Reference |  | Reference |  |
| Moderate delay | 1.283(1.189-1.384) | ＜0.001 | **1.259(1.168-1.358)** | ＜0.001 |
| Long delay | 1.572(1.447-1.709) | ＜0.001 | **1.693(1.560-1.838)** | ＜0.001 |

HR: hazard ratio; 95%CI: 95% confidential interval

|  |
| --- |

**Supplementary Table 3.** Subgroup analyses for the association of the association between demographic factors, clinical characteristics, and cancer-specific survival (CSS) in patients with esophageal cancer.

| **Characteristics** | **Brief delay** | **Moderate delay** | **Long delay** | **P-value** |
| --- | --- | --- | --- | --- |
| **Total number** |  |  |  |  |
| **Sex** |  |  |  |  |
| Male | Reference | **1.310(1.201-1.428)** | **1.578(1.437-1.733)** | ＜0.001 |
| Female | Reference | **1.215(1.034-1.427)** | **1.575(1.315-1.885)** | ＜0.001 |
| **Age group (years)** |  |  |  |  |
| ≤44 | Reference | 1.374(0.874-2.160) | 1.301(0.800-2.115) | 0.375 |
| 45-54 | Reference | **1.470(1.164-1.857)** | **1.759(1.376-2.247)** | ＜0.001 |
| 55-64 | Reference | 1.121(0.970-1.296) | **1.317(1.126-1.540)** | ＜0.001 |
| 65-74 | Reference | **1.394(1.211-1.603)** | **1.770(1.517-2.065)** | ＜0.001 |
| ≥75 | Reference | **1.303(1.129-1.505)** | **1.761(1.499-2.068)** | ＜0.001 |
| **Marital status** |  |  |  |  |
| Single | Reference | **1.245(1.114-1.390)** | **1.664(1.475-1.878)** | ＜0.001 |
| Couple | Reference | **1.324(1.191-1.471)** | **1.523(1.358-1.708)** | ＜0.001 |
| **Household income** |  |  |  |  |
| Low level | Reference | 1.182(0.951-1.470) | **1.478(1.170-1.866)** | ＜0.001 |
| Middle level | Reference | **1.359(1.213-1.523)** | **1.668(1.472-1.889)** | ＜0.001 |
| High level | Reference | **1.236(1.100-1.388)** | **1.502(1.323-1.705)** | ＜0.001 |
| **Living areas** |  |  |  |  |
| Metropolitan areas | Reference | **1.293(1.192-1.402)** | **1.580(1.446-1.727)** | ＜0.001 |
| Nonmetropolitan areas | Reference | 1.242(0.994-1.552) | **1.555(1.226-1.971)** | ＜0.001 |
| **Primary site** |  |  |  |  |
| Upper third of esophagus | Reference | **1.410(1.033-1.925)** | **1.664(1.191-2.325)** | 0.010 |
| Middle third of esophagus | Reference | **1.451(1.193-1.765)** | **1.977(1.594-2.453)** | ＜0.001 |
| Lower third of esophagus | Reference | **1.258(1.140-1.388)** | **1.488(1.337-1.657)** | ＜0.001 |
| Other site | Reference | **1.234(1.034-1.472)** | **1.537(1.269-1.862)** | ＜0.001 |
| **Diferentiation** |  |  |  |  |
| Highly diferentiated | Reference | 1.107(0.810-1.514) | **1.641(1.140-2.363)** | 0.004 |
| Moderately diferentiated | Reference | **1.365(1.213-1.536)** | **1.574(1.382-1.793)** | ＜0.001 |
| Poor diferentiated | Reference | **1.229(1.105-1.368)** | **1.550(1.381-1.739)** | ＜0.001 |
| Undiferentiated | Reference | 1.406(0.661-2.993) | 1.657(0.757-3.630) | 0.374 |
| **Stage** |  |  |  |  |
| Ⅰ | Reference | 1.086(0.888-1.327) | **1.299(1.027-1.644)** | 0.052 |
| Ⅱ | Reference | **1.373(1.152-1.637)** | **1.629(1.323-2.006)** | ＜0.001 |
| Ⅲ | Reference | 1.066(0.917-1.240) | 1.167(0.979-1.391) | 0.161 |
| Ⅳ | Reference | **1.474(1.308-1.662)** | **1.849(1.631-2.097)** | ＜0.001 |
| **Histology** |  |  |  |  |
| Squamous cell carcinoma | Reference | **1.445(1.276-1.637)** | **1.792(1.562-2.056)** | ＜0.001 |
| Adenocarcinoma | Reference | **1.202(1.083-1.333)** | **1.424(1.272-1.593)** | ＜0.001 |
| Other | Reference | 1.223(0.938-1.594) | **1.700(1.272-2.272)** | ＜0.001 |
| **Radiotherapy** |  |  |  |  |
| Yes | Reference | **1.300(1.192-1.417)** | **1.622(1.474-1.784)** | ＜0.001 |
| No | Reference | **1.300(1.105-1.529)** | **1.507(1.269-1.788)** | ＜0.001 |
| **Chemotherapy** |  |  |  |  |
| Yes | Reference | **1.189(1.091-1.296)** | **1.363(1.241-1.498)** | ＜0.001 |
| No | Reference | **1.498(1.273-1.763)** | **2.424(2.032-2.892)** | ＜0.001 |

95%CI: 95% confidential interval
